# Association between chronic viral hepatitis infection and breast cancer risk: a nationwide population-based case-control study

**DOI:** 10.1186/1471-2407-11-495

**Published:** 2011-11-24

**Authors:** Fu-Hsiung Su, Shih-Ni Chang, Pei-Chun Chen, Fung-Chang Sung, Chien-Tien Su, Chih-Ching Yeh

**Affiliations:** 1 School of Public Health, College of Public Health and Nutrition, Taipei Medical University, 250 Wu-Hsing Street, 11031 Taipei, Taiwan; 2 Department of Family Medicine, Pojen General Hospital, 66 Kwang Fu N. Rd, 101 Taipei, Taiwan; 3 The Ph.D. Program for Cancer Biology and Drug Discovery, China Medical University, No.91, Hsueh-Shih Road, 40402 Taichung, Taiwan; 4 Institute of Biomedical Sciences, Academia Sinica, 128, Sec. 2, Academia Rd, 115 Taipei, Taiwan; 5 Management Office for Health Data, China Medical University Hospital, No.91, Hsueh-Shih Road, 40402 Taichung, Taiwan; 6 Institute of Clinical Medical Science, China Medical University, No.91, Hsueh-Shih Road, 40402 Taichung, Taiwan; 7 Department of Health Risk Management, China Medical University, No.91, Hsueh-Shih Road, 40402 Taichung, Taiwan; 8 Department of Public Health, China Medical University, No.91, Hsueh-Shih Road, 40402 Taichung, Taiwan; 9 Department of Family Medicine, Taipei Medical University Hospital, 252 Wu-Hsing Street, 11031 Taipei, Taiwan

## Abstract

**Background:**

In Taiwan, there is a high incidence of breast cancer and a high prevalence of viral hepatitis. In this case-control study, we used a population-based insurance dataset to evaluate whether breast cancer in women is associated with chronic viral hepatitis infection.

**Methods:**

From the claims data, we identified 1,958 patients with newly diagnosed breast cancer during the period 2000-2008. A randomly selected, age-matched cohort of 7,832 subjects without cancer was selected for comparison. Multivariable logistic regression models were constructed to calculate odds ratios of breast cancer associated with viral hepatitis after adjustment for age, residential area, occupation, urbanization, and income. The age-specific (<50 years and ≥50 years) risk of breast cancer was also evaluated.

**Results:**

There were no significant differences in the prevalence of hepatitis C virus (HCV) infection, hepatitis B virus (HBV), or the prevalence of combined HBC/HBV infection between breast cancer patients and control subjects (*p *= 0.48). Multivariable logistic regression analysis, however, revealed that age <50 years was associated with a 2-fold greater risk of developing breast cancer (OR = 2.03, 95% CI = 1.23-3.34).

**Conclusions:**

HCV infection, but not HBV infection, appears to be associated with early onset risk of breast cancer in areas endemic for HCV and HBV. This finding needs to be replicated in further studies.

## Background

Breast cancer is one of the most common cancers and the leading cause of death in women worldwide [1]. Over the past several decades, the incidence of breast cancer has increased globally [2,3], with the greatest increase occurring in Asian countries [2,4,5]. Risk factors for breast cancer include benign breast disease, fertility, obesity, particularly after menopause, familial and genetic factors, and oral contraceptive use [6-9]. The etiology and progression of breast cancer remain incompletely understood; therefore, novel routes of disease pathogenesis are important to consider.

Richardson proposed that breast cancer risk may be associated with late exposure to common viruses [10]. Zur Hausen stated that approximately 19% of the global cancer burden can be linked to five infectious agents, namely Epstein-Barr virus (EBV), human Papillomaviruses (HPV), hepatitis B virus (HBV), hepatitis C virus (HCV), and helicobacter pylori [11]. His findings, therefore, suggest that viruses may be considered as a potential cause of breast cancer [11-13]. Yasui et al. provided supporting evidence for the hypothesis that "delayed" exposure to Epstein-Barr virus is a risk factor for breast cancer [14]. Mazouni et al. also suggested that Epstein-Barr virus is a marker of biological aggressiveness in breast cancer [15]. In addition, de Villiers and his colleagues demonstrated the occurrence of HPV in nipple and areolar tissues in patients with breast carcinoma [12]. Besides the well established association with primary liver cancer, HCV infection has been associated with other neoplasms including non-Hodgkin lymphoma [16], and smoking and alcohol-related cancers, such as cancers of the pancreas, lung and kidney, and the oropharygeal cancer [17]. In the same token, HBV infection also has been associated with pancreas cancer [18], intrahepatic cholangiocaricinoma and non-Hodgkin lymphoma [19,20]. Whether there is a potential link between chronic HCV or HBV infection and the risk of developing breast cancer has yet to be well investigated.

In Taiwan, the incidence of breast cancer has increased annually by 8% since 2003 [21]. In addition, the peak age for breast cancer is between 40 years and 50 years in Taiwan whereas the peak age in Western countries is between 60 years and 70 years [2]. Taiwan is a country in which HBV infection is endemic and a country with a high prevalence of HCV [22]. The fact that Taiwan is endemic for viral hepatitis and has a high prevalence of breast cancer provides an excellent setting in which to study the association of these 2 entities. Therefore, we used a nationwide population-based dataset to assess the possible association between chronic viral hepatitis infection and breast cancer as well as the possibility that chronic viral hepatitis is a risk factor for early onset breast tumors in Taiwan.

## Materials and methods

### Data sources

Data used in this study were retrieved from the National Health Insurance Research Database (NHIRD), which is maintained by the National Health Research Institute (NHRI), Taiwan. The single-payer National Health Insurance (NHI) program in Taiwan was initiated in March, 1995 to provide comprehensive and affordable health care to all of the island's residents. By the end of 1996, this insurance program covered more than 96% of the population and has contracted with 97% of hospitals and clinics in the country [23]. The NHRI randomly selected data from the insured population to establish a representative sub-dataset comprised of 1 M insured enrollees during the period 1996-2000. There is no significant difference in the distribution of gender, age, or average payroll-related insurance payment between the individuals in the NHRID and the original medical claims for all enrollees under the NHI program. The data files are linkable through an encrypted but unique personal identification number and thus provide patient-level information on demographic characteristics, medical history, registry of medical facilities, details of inpatient orders, ambulatory care visits, dental services, and prescriptions. Diagnoses are coded according to the International Classification of Disease, Ninth revision (ICD-9-CM). As the dataset was released with de-identified secondary data for public research purposes, the study was exempt from full review by the Institutional Review Board.

### Study sample

Patients with newly diagnosed breast cancer (ICD-9-CM code 174 and 175) during the period 2000-2008 were identified from the registry for Catastrophic Illness Patients Database. The insurance coverage for catastrophic illnesses is the extension of the Taiwan's NHI to protect people with serious disease against catastrophic financial burden and subsequent impoverishment. Breast cancer was placed in the category of NHI-defined "Catastrophic Illnesses", and the NHI covers the treatment costs incurred by this disease. In this study, our purpose was to observe mainly the association between chronic viral hepatitis (HBV or HCV) and breast cancer. The etiologies of other types chronic hepatitis, such as autoimmune, chemical, alcoholic-related hepatitis, as well as non-alcoholic fatty liver disease were not included as our study subjects. For HBV infection recorded in the database, the presence of HBs Ag was the major serum marker. Patients with the history of HIV were excluded from the present study to minimize the inclusion of occult HBV infection (the persistence of hepatitis B virus (HBV) in hepatitis B surface antigen (HBs Ag) negative) among patients with the HBV/HIV coinfection [24]. Hence, we excluded patients with a history of HIV (ICD-9-CM 042, 043, 044, and V08), chronic hepatitis (ICD-9-CM 070.9, 571.4, 571.8, 571.9, and 573.3) without mentioning hepatitis B (ICD-9-CM 070.2, 070.3, and V02.61) or hepatitis C (ICD-9-CM 070.41, 070.44, 070.51, 070.54, and V02.62) infection. After excluding two cases of HIV and 468 subjects with chronic hepatitis without mentioning hepatitis B or hepatitis C, 1958 subjects with breast cancer were enrolled in this study.

Control subjects were randomly extracted from the remaining subjects in the database with an identical exclusion criterion to cases. The comparison group comprised randomly selected age- and sex-matched individuals without a history of breast cancer. The control to patient ratio was 4:1. The age of each study subject was based on the difference in time between the index date and the date of birth. Originally, 434,659 female subjects were retrieved from the NHIRD. After excluding 9,738 subjects with cancers other than breast cancer, 260 cases with HIV, and 69,756 subjects with chronic hepatitis other than hepatitis B or hepatitis C, 354,905 subjects were eligible for the selection of control subjects. Finally, a total of 7,832 controls were enrolled in this study.

### Statistical analysis

We compared the distributions of demographic characteristics, including age, occupation, urbanization level, and personal income between breast cancer patients and control subjects using Chi-square tests. Potential confounders included in this study were monthly income, level of urbanization (4 levels with Level 1 referring to the "most urbanized" and Level 4 referring to the "least urbanized" communities), and the geographical location of the community in which the patient resided (northern, central, eastern, and southern Taiwan). We selected NT $15,000 and NT $30,000 as the income level cutoff points in Taiwan. Multivariable logistic regression analysis was conducted to assess the association of viral hepatitis with the risk of breast cancer, after adjusting for variables that were significantly related to breast cancer from the prior Chi-square analyses. The age-specific odds ratios of breast cancer associated with viral hepatitis were also examined by two individual logistic regression models (one for age <50 years and the other one for age ≥50 years). To address the hypothesis of the presence of an association between HCV and early onset breast cancer, interaction between HCV status and age of developing breast cancer was tested by a logistic regression analysis. All analyses were performed by SAS statistical software (version 9.1 for Windows; SAS Institute, Inc., Cary, NC, USA), and the significance level was set at 0.05.

## Results

### Characteristics of the study subjects

Table 1 compares the distributions of demographic characteristics between the patient group and the control group. Most subjects were in the 40-60-year age group (64.3% in the patient group and the control group). Breast cancer patients were more likely than control subjects to have white-collar occupations (55.4% vs. 51.3%, *p *= 0.001), live in areas with the highest urbanization level (34.4% vs. 31.7%, *p *= 0.03), and have higher incomes (15.7% vs. 12.6%, *p *= 0.0004).

**Table 1:** Demographic characteristics between breast cancer patients and non-breast cancer subjects in 2000-2008

Variables	Total *N *= 9790		Breast cancer				p-value*
				
			No *N *= 7832		Yes *N *= 1958		
		
	n	(%)	n	(%)	n	(%)	
Age, years							1.00

20-29	145	(1.5)	116	(1.5)	29	(1.5)	

30-39	1305	(13.3)	1044	(13.3)	261	(13.3)	

40-49	3630	(37.1)	2904	(37.1)	726	(37.1)	

50-59	2665	(27.2)	2132	(27.2)	533	(27.2)	

60-69	1205	(12.3)	964	(12.3)	241	(12.3)	

≥ 70	840	(8.6)	672	(8.6)	168	(8.6)	

Occupation							0.001

White collar	5098	(52.1)	4014	(51.3)	1084	(55.4)	

Blue collar	3538	(36.1)	2898	(37.0)	640	(32.7)	

Others	1154	(11.8)	920	(11.8)	234	(12.0)	

Urbanization level							0.03

1	3154	(32.2)	2480	(31.7)	674	(34.4)	

2	2928	(29.9)	2335	(29.8)	593	(30.3)	

3	1675	(17.1)	1351	(17.3)	324	(16.6)	

4	2032	(20.8)	1666	(21.3)	366	(18.7)	

Region							0.24

North	4762	(48.7)	3782	(48.3)	980	(50.1)	

Central	1880	(19.2)	1495	(19.1)	385	(19.7)	

South	2330	(23.8)	1888	(24.1)	442	(22.6)	

East and Islands	817	(8.4)	667	(8.5)	150	(7.7)	

Income							0.0004

< 15,000	3116	(31.8)	2481	(31.7)	635	(32.4)	

15,000-29,999	5381	(55.0)	4365	(55.7)	1016	(51.9)	

≥ 30,000	1293	(13.2)	986	(12.6)	307	(15.7)	

Urbanization level: 1 Indicate the highest level of urbanization and 4 the lowest

*Chi-square test

Table 2 shows the prevalence of viral hepatitis B and C in the study subjects. Although the patient group tended to have higher proportions of HCV (2.9% vs.2.3%), HBV (6.3% vs.6.1%) or both (1.0% vs.0.9%), there were no significant differences in the prevalence of those infections between patients and controls (*p *= 0.48).

**Table 2:** Comparison of hepatitis prevalence between breast cancer patients and non-breast cancer subjects in 2000-2008

Viral hepatitis B & C	Total *N *= 9790		Breast cancer					
					
			No *N *= 7832		Yes *N *= 1958			
	
	n	(%)	n	(%)	n	(%)	OR^a^	(95% CI)
No	8862	(90.5)	7102	(90.7)	1760	(89.9)	1.00	(reference)

HCV	234	(2.4)	178	(2.3)	56	(2.9)	1.28	(0.95-1.75)

HBV	603	(6.2)	480	(6.1)	123	(6.3)	1.05	(0.85-1.28)

HBV + HCV	91	(0.9)	72	(0.9)	19	(1.0)	1.08	(0.65-1.80)

^a^Adjusted for area, occupation, urbanization, and income

### Overall risk and age-specific risk of breast cancer

The logistic regression analysis showed that viral hepatitis was not significantly associated with breast cancer, although the risk was greater for subjects with HCV (adjusted OR = 1.28, 95% CI = 0.95-1.78) than for those with HBV (adjusted OR = 1.05, 95% CI = 0.85-1.28) or both (adjusted OR = 1.08, 95% CI = 0.65-1.80) (Table 2).

However, the multivariable regression model revealed that HCV patients aged less than 50 years had a significant 2.03-fold higher risk of breast cancer (OR = 2.03, 95% CI = 1.23-3.34) (Table 3). The interaction between HCV status and age (<50 years and ≥50 years) of developing breast cancer was statistically significant (*p *= 0.019) (not shown in Table).

**Table 3:** Odds ratios and 95% confidence interval of breast cancer associated with viral hepatitis by age

Viral hepatitis B & C	Age < 50 years				Age ≥ 50 years			
	
	Cases	Controls	OR^a^	(95% CI)	Cases	Controls	OR^a^	(95% CI)
No	910	3722	1.00	(reference)	850	3380	1.00	(reference)
HCV	24	48	2.03	(1.23-3.34)*	32	130	1.00	(0.67-1.49)
HBV	72	267	1.10	(0.84-1.45)	51	213	0.97	(0.71-1.33)
HBV + HCV	10	27	1.59	(0.77-3.31)	9	45	0.83	(0.40-1.71)

^a^Adjusted for area, occupation, urbanization, and income

*p < 0.01 based on Wald tests

## Discussion

In this large-scale, population-based study in an area where both viral hepatitis and breast cancer are prevalent, we found no significant association between breast cancer risk and HBV or HCV seropositivity; however, age-stratification analysis showed that HCV patients aged less than 50 years had a significant two-fold greater risk of breast cancer.

Although breast cancer is mainly a postmenopausal disease, breast cancer in younger females often shows more aggressive clinical features and worse prognosis [3]. Studies have shown that high-risk and low-risk tumors present different age distributions, suggesting that breast cancer comprises a mixture of two different disease processes [3,25]. The epidemiology of breast cancer in women living in Asia differs from that in women living in North America or Europe. The peak incidence age is between 40 years and 50 years among Asian women, whereas in the United States and Europe, it peaks among women in the sixth decade of life [2,5,26,27]. This variation in peak incidence age may be due to multiple factors, including geographic variation, racial/ethnic background, genetic variation, lifestyle, environmental factors, socioeconomic status, utilization of screening mammography, stage of disease at diagnosis, and the availability of appropriate care [1,21]

In 1997, Richardson proposed that breast cancer risk is associated with late exposure to common viruses [10]. Since then, numerous studies have shown that EBV, HPV, and cytomegalovirus infections are associated with the development of breast cancer [12,14,15,28,29].

Few studies, however, have evaluated the relationship between HCV or HBV infection and breast tumor development. A higher prevalence of HCV has been observed in elderly patients with tumors of the colon/rectum, prostate, breast, bladder, or kidney [30-32] as well as in elderly patients with hepatocarcinoma [33] or non-Hodgkin lymphoma [16,19]. Malaguarnera et al. reported that HCV RNA was detectable in sera in 11% of breast cancer patients and in 6.6% of control subjects, although there was no significant difference between the two groups [30]. Bruno et al. recently reported that HCV infection most likely plays an important role in the development of hepatocellular carcinoma as well as breast cancer [32]. In contrast, the results of a recent case-control study conducted by Larrey and his colleagues suggest that chronic HCV infection is not a strong promoter of breast carcinoma in adult females of any age [31]. However, a relatively small number of breast cancer patients in the control group may have masked the true significance of the infection after age stratification. Similarly, we found that chronic HCV infection was not significantly associated with breast cancer; however, after stratification by age (sub-grouped into <50 years and ≥50 years), chronic HCV infection was more prevalent among patients with early-onset breast cancer than among those with late-onset of the disease. This finding also suggests that breast cancer may comprise a mixture of two different disease processes, and that HCV may act as a promoter of carcinogenesis in young patients. Further studies are needed.

The mechanism responsible for the oncogenetic role of HCV is not well understood, but it involves immunity and autoimmunity disorders [30,34,35]. Mechanisms of direct and indirect carcinogenesis by which infections may contribute to cancer development have been suggested. Immunosuppression or induction of reactive oxygen species via inflammatory reactions have been suggested as the two most prominent pathways for indirect infectious carcinogens. Unlike the immunosuppressive mechanism induced by HIV, the mechanism by which Hepatitis B and C viruses contribute to cancer still remains obscure [11].

In this study, neither infection with HBV alone nor HBV/HCV coinfection was correlated with breast cancer. HBV and HCV are both hepatotropic viruses. Their coinfection is associated with clinically and histologically more severe liver disease and higher risk of the development of hepatocellular carcinoma [22,36]. However, HCV infection has been reported to predispose patients to extrahepatic disorders involving renal, dermatologic, hematologic, and rheumatologic systems as well as autoimmune abnormalities [37-39]. Extrahepatic manifestations may result from immunologic triggered mechanisms as well as virus invasion and replication that affect extrahepatic tissues and organs. Only HCV has the lymphotropic character that is assumed to be the cause of HCV-associated extrahepatic manifestation [40]. That may explain why we found that HCV, but not HBV, was associated with breast cancer. Further prospective cohort studies are required to verify this result.

This study has several important limitations. First, some hepatitis infected patients without obvious clinical symptoms might not receive medical services. As a result, some hepatitis infected subjects are included in the control group. However, if viral hepatitis infection is associated causally with breast cancer, this misclassification may lead the estimated HRs toward the null and further strengthen our finding. Therefore, we are confident of the positive association between HCV and early-onset breast cancer. Second, using ICD codes to select patients with diagnoses of breast cancer, HBV infection, HCV infection, or other comorbid medical conditions may be less accurate than selecting patients in a clinical setting. However, the BNHI randomly samples a fixed percentage of claims from every hospital and randomly interviews patients and reviews charts each year to verify the validity of diagnoses and the quality of care. Patients with confirmed breast cancer in Taiwan are placed in the category of "Catastrophic Illness", and the BNHI covers the treatment costs incurred by those patients. Hence, the diagnosis is relatively accurate and the patients are representative of the population in Taiwan. Third, the majority of the residents in Taiwan are of Chinese ethnicity. The ability to generalize the results to other racial/ethnic groups is unclear given that the transmission route of viral hepatitis infection in Chinese might not be completely the same as that in other ethnic groups. Finally, important variables that might be related to both hepatitis and breast cancer were unavailable in the insurance claims database, including family cancer history, body mass index, environmental exposure, nutritional status, physical activity level, hormone receptor status of breast cancer, cigarette smoking, and alcohol consumption. Therefore, we cannot rule out some of the potential confounding effects associated with these factors.

## Conclusions

In summary, this population-based study suggests that chronic HCV infection is associated with early-onset breast cancer and that chronic HBV infection is not associated with breast cancer. Further studies are needed to apply our finding to other regions or races and to clarify the underlying pathophysiological mechanisms behind the association of chronic viral hepatitis with breast cancer.

## Abbreviations

CI: Confidence interval; EBV: Epstein-Barr virus; HBV: Hepatitis B virus; HCV: Hepatitis C virus; HPV: Human papillomaviruses; HIV: Human immunodeficiency virus; ICD-9-CM: International classification of disease diagnoses, ninth revision; NHI: National Health Insurance; NHIRD: National Health Insurance Research Database; NT: New Taiwanese Dollar; OR: Odds ratio.

## Competing interests

The authors declare that they have no competing interests.

## Authors' contributions

FHS helped to design, analyze and interpret the data and drafted the manuscript. SNC, PCC, FCS, and CTS helped to perform statistical analysis and data interpretation. CCY conceived of the study, participated in study design, analysis, and interpretation, and critically revised the manuscript. All authors read and approved the final manuscript.

## Pre-publication history

The pre-publication history for this paper can be accessed here:

http://www.biomedcentral.com/1471-2407/11/495/prepub
